# Screening of a Combinatorial Library of Organic Polymers for the Solid-Phase Extraction of Patulin from Apple Juice

**DOI:** 10.3390/toxins9050174

**Published:** 2017-05-20

**Authors:** Cristina Giovannoli, Giulia Spano, Fabio Di Nardo, Laura Anfossi, Claudio Baggiani

**Affiliations:** Laboratory of Bioanalytical Chemistry, Department of Chemistry, University of Torino, Via Giuria 5, Torino 10125, Italy; giulia.spano@unito.it (G.S.); fabio.dinardo@unito.it (F.D.N.); claudio.baggiani@unito.it (C.B.)

**Keywords:** patulin, mycotoxin, solid-phase extraction, combinatorial library

## Abstract

Patulin is a water-soluble mycotoxin produced by several species of fungi. Governmental bodies have placed it under scrutiny for its potential negative health effects, and maximum residue limits are fixed in specific food matrices to protect consumers’ health. Confirmatory analysis of patulin in complex food matrices can be a difficult task, and sample clean-up treatments are frequently necessary before instrumental analyses. With the aim of simplifying the clean-up step, we prepared a 256-member combinatorial polymeric library based on 16 functional monomers, four cross-linkers and four different porogenic solvents. The library was screened for the binding towards patulin in different media (acetonitrile and citrate buffer at pH 3.2), with the goal of identifying polymer formulations with good binding properties towards the target compound. As a proof of concept, a methacrylic acid-co-pentaerithrytole tetraacrylate polymer prepared in chloroform was successfully used as a solid-phase extraction material for the clean-up and extraction of patulin from apple juice. Clean chromatographic patterns and acceptable recoveries were obtained for juice spiked with patulin at concentration levels of 25 (64 ± 12%), 50 (83 ± 5.6%) and 100 μg L^−1^ (76 ± 4.5%). The within-day and between-day reproducibility evaluated at a concentration level of 25 μg L^−1^ were 5.6 and 7.6%, respectively.

## 1. Introduction

Patulin (4-hydroxy-4H-furo[3,2-c]pyran-2[6H]-one) is a water-soluble ylidenebutenolide mycotoxin produced by several *Aspergillus* and *Penicillium* species of fungi whose presence is considered common in fruit- and vegetable-based products, especially apples and apple-based food and beverages. Although patulin was first identified as a compound with antibiotic properties, and was thus considered interesting for pharmaceutical applications [1,2], it has been placed under surveillance for its potential negative health effects. Several studies related to its suspected mutagenic, teratogenic and carcinogenic activities have confirmed its ability to inhibit in vitro and in vivo DNA, RNA, and protein synthesis [3,4,5]. As a consequence, in order to protect consumers’ health, several government agencies have posed maximum residue limits (MRLs) for patulin in specific food matrices. The European Union regulation has set an MRL of 50 μg kg^−1^ in apple juice and as a juice ingredient in different beverages, 25 μg kg^−1^ in solid apple products and 10 μg kg^−1^ in apple products intended for infants and young children [6].

Confirmatory analyses for the presence of residues of patulin in food products require affordable instrumental analytical methods. HPLC with UV or mass spectrometry detection appears to be the most affordable and most used approach [7,8,9,10]. Since the direct detection of patulin in complex food matrices is a very difficult task, sample clean-up treatments are generally required before instrumental analyses [11,12,13]. More generally, the use of preliminary clean-up protocols can be considered a rather common requirement in analytical methods developed for the determination of different contaminants in food matrices. In recent years to simplify clean-up steps, molecularly imprinted polymers as highly selective synthetic materials have been proposed for the extraction and pre-concentration of different mycotoxins from different food samples by using as a template the mycotoxin itself, and more often a mimic molecule [14,15,16]. Several papers have recently described synthetic polymers prepared by polymerization without the use of the template molecule, but nevertheless capable of good selectivity and binding properties towards deoxynivalenol, aflatoxin B1, and ochratoxin A [17,18,19,20]. These studies are based on the virtual screening of an in silico library of functional monomers and cross-linkers for the binding to a target molecule. Similarly, it is plausible that binding polymeric materials can be obtained by directly screening a library of organic polymers characterized by a high degree of molecular diversity, with the advantage of traditional molecular imprinting that does not require the use of a toxic and expensive template molecule like patulin. 

In this paper, the preparation of a 256-member combinatorial polymeric library based on the use of 16 functional monomers, four cross-linkers and four different porogenic solvents was described. Then, the library was screened for the binding to patulin in different experimental conditions and the organic polymer that showed the best binding properties was selected as a solid-phase extraction material for the extraction of patulin from apple juice. 

## 2. Results and Discussion

### 2.1. Synthesis and Screening of the Polymeric Combinatorial Library

To ensure a large degree of molecular diversity, we combined in a 256-members polymeric library functional monomers, cross-linkers, and porogenic solvents largely different each other in chemical and physical properties. Neutral polar (acrylamide, acrylonitrile, 4-acryloylmorpholine, *N*,*N*-dimethylacrylamide, 2-hydroxyethylmethacrylate, monomethoxypolyethylenglycol 400 methacrylate, *N*-vinylpyrrolidone), neutral hydrophobic (methylacrylate, styrene), acid (ethylenglycol methacrylate phosphate, methacrylic acid,) and basic (allylamine, *N*,*N*-diethylaminoethylmethacrylate, *N*,*N*-dimethylaminoethylmethacrylate, 1-vinylimidazole, 4-vinylpyridine) compounds were used as functional monomers. Cross-linkers were selected according to the number of possible polymerizable groups and to their increasing hydrophobicity: two groups (ethylene dimethacrylate, logP = 3.26; glycerol dimethacrylate, logP = 2.59), three groups (trimethylolpropane trimethacrylate, logP = 5.42) and four groups (pentaerithrytole tetraacrylate, logP = 4.77). Porogenic solvents were selected in order to represent different types of organic solvent: with aromatic (toluene), hydrophobic (chloroform) and hydrophilic (acetonitrile and tetrahydrofurane) features. In fact, it should be considered that, apart from effect due to the presence of different functional monomers, diversity in binding behavior may potentially raise both from the use of different acrylic cross-linkers, which may indirectly influence the hydrophobic character of the polymeric surface, and from the use of different porogenic solvents, resulting in polymers with markedly different surface morphologies. 

From the experimental results reported in Table 1, Table 2, Table 3 and Table 4, we can observe that the environment in which the library screening was performed deeply influences the retention behavior of patulin. In fact, when the screening is performed in an acidic buffer, patulin is completely retained by most of the polymers (200/256 with B/T ≥ 0.95), and even the less binding ones show high B/T (bound-to-total) values, as in the case of the weakest binding polymer (MMA + GDMA in toluene) that shows a B/T value of 0.63. These results were not unforeseen, because any weak hydrophobic interactions between patulin and the hydrophobic polymer surface should be strengthened by the presence of aqueous buffers.

**Figure 1:**
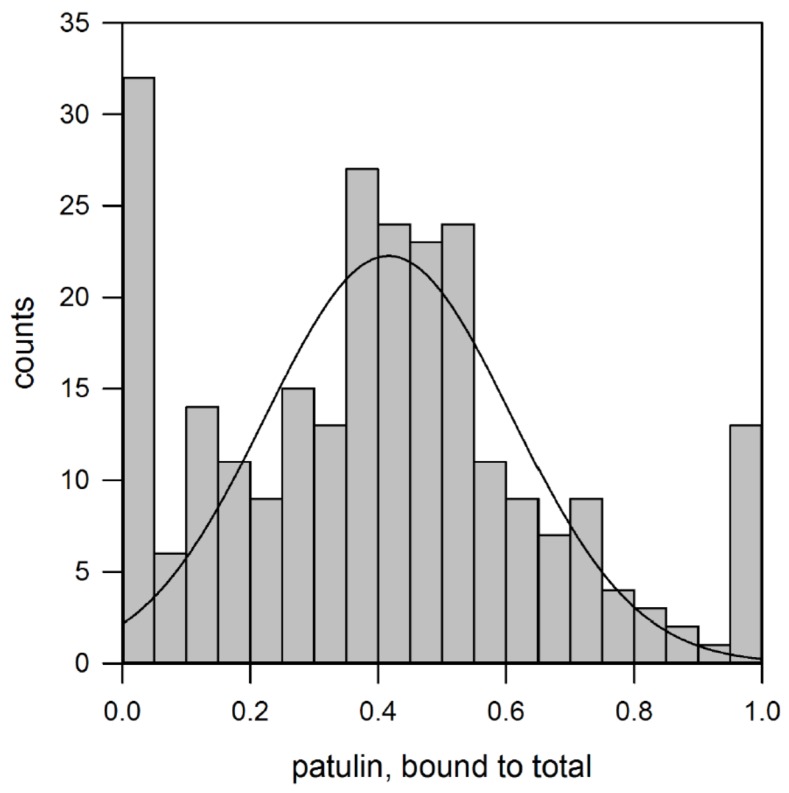
Distribution of patulin binding capacity. The continuous curve indicates the normal distribution interpolated by the experimental results with the exclusion of extreme values (B/T ≤ 0.05 and B/T ≥ 0.95).

On the contrary, when the screening is performed in acetonitrile the results are completely different. In fact, patulin is completely retained only by a small number of polymers (13/256 with B/T ≥ 0.95), while a greater number of them (33/256 with B/T ≤ 0.05) show no retention. As reported in Figure 1, when excluding the extreme values (B/T ≤ 0.05 and B/T ≥ 0.95), the polymer library shows binding capacities scattered throughout the whole spectra of B/T values, with a normal distribution characterized by a mean value of B/T = 0.42 ± 0.19. This binding distribution and the number of polymers with “extreme” behavior (completely or no retention at all) can be explained by considering that acetonitrile is a moderately polar, aprotic solvent. In fact, for most of the polymers, the use of acetonitrile does not interfere with the polar hydrogen bond between the patulin and the functional groups on the polymeric surface. Conversely, the presence of the organic solvent leads to the disruption of the hydrophobic interactions with a significant drop of the patulin retention with respect to the aqueous environment.

**Figure 2:**
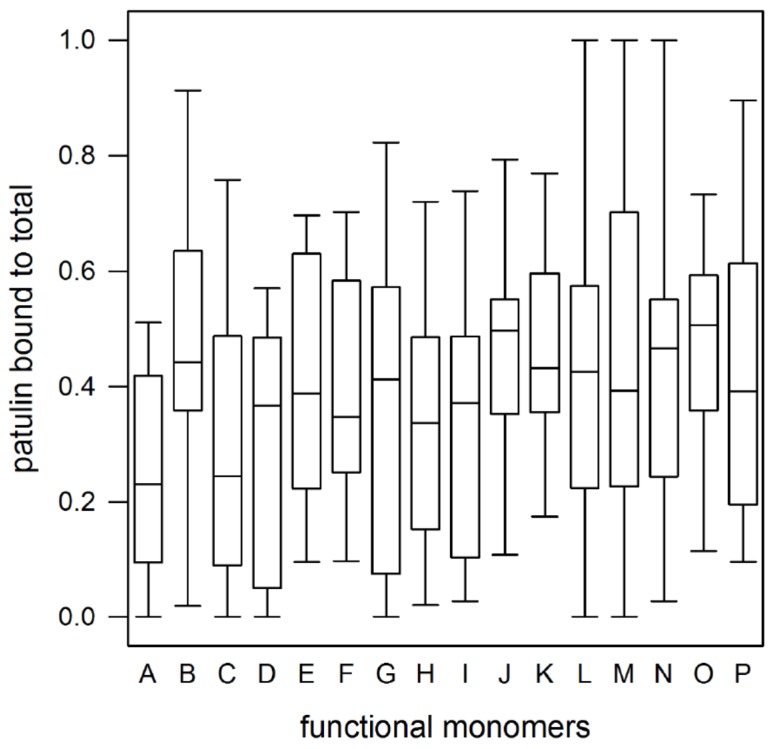
Patulin binding measured for polymers whose formulation contains the same functional monomer. A: MAA; B: DMAEM; C: EGMP; D: ALA; E: DEAEM; F: 4VP; G: VIM; H: AM; I: AN; J: DMA; K: HEMA; L: MMA; M: STY; N: ACM; O: VPO; P: PEGMA. Upper and lower solid boundaries of the boxes indicate the 25th and 75th percentiles; the lines within the boxes mark the medians (50th percentiles); the whiskers above and below the boxes indicate the 90th and 10th percentiles of the data.

An in-depth statistical analysis performed by ANOVA tests on ranks was conducted to compare B/T values related to groups of polymers prepared with the same porogenic solvent, cross-linker or functional monomer. The overall results show very limited differences between data groups. In fact, polymers prepared with the same porogenic solvent or cross-linker do not show any statistically significant difference between data groups (*p* < 0.05), with the only exception of polymers prepared with the cross-linker TRIM, which retain patulin better than the same polymers prepared with PETRA (*p* = 0.007). As regards the effect of the functional monomers, in Figure 2a statistically significant difference among data groups can be observed between polymers prepared with MAA and polymers prepared with some basic (DEAEM, *p* = 0.007; 4VP, *p* = 0.041), neutral polar (DMA, *p* = 0.06; HEMA, *p* = 0.006; ACM, *p* = 0.02; VPO, *p* = 0.003; PEGMA, *p* = 0.044) or hydrophobic (MMA, *p* = 0.04; STY, *p* = 0.027) monomers. In conclusion, it is not possible to identify a clear trend in patulin retention which will be directly related to the different polymer formulation. This can be ascribed to the different effects of functional monomers, cross-linkers and porogenic solvents that combine together in a very complex manner to determine the overall binding properties of the resulting polymer. 

### 2.2. Solid-Phase Extraction of Apple Juice 

The first part of the experimental work was focused on the screening of the polymer libraries in order to find out the polymerization mixtures with good binding properties towards patulin in acidic buffer (B/T ≥ 0.95) and almost negligible binding properties in acetonitrile (B/T ≤ 0.05). From the experimental results reported in Table 1, Table 2, Table 3 and Table 4, we identified 25 polymers with the desired properties.

As a proof of concept, we randomly selected and used one of them, the methacrylic acid-*co*-pentaerithrytole tetraacrylate polymer prepared in chloroform (MAA + PETRA/chloroform), to set up a solid-phase extraction procedure of patulin from apple juice. 

**Figure 3:**
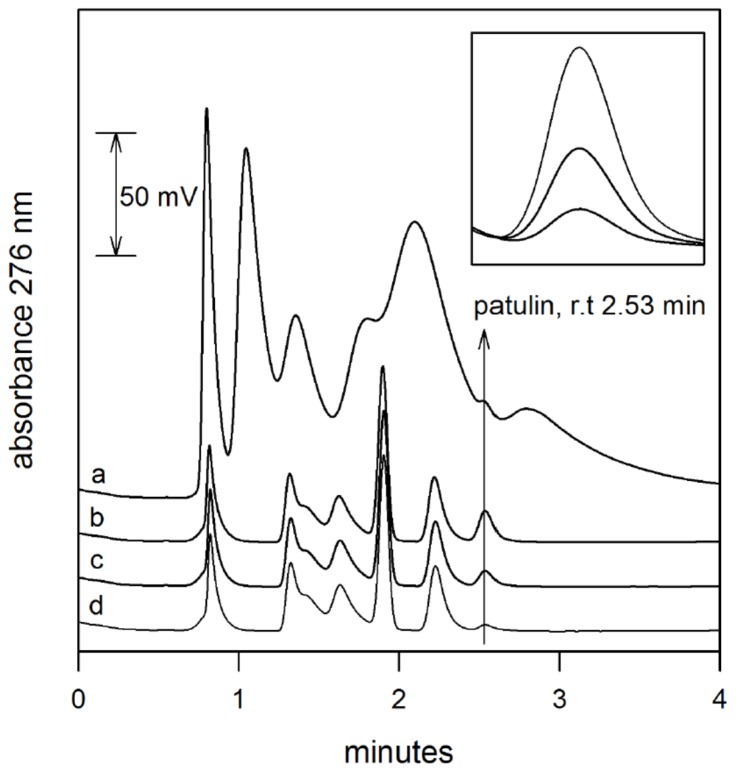
RP-HPLC of apple juice spiked at 100 μg L^−1^ (a) and apple juice spiked at 100 (b), 50 (c) and 25 (d) μg L^−1^ after solid-phase extraction on a cartridge packed with methacrylic acid-*co*-pentaerithrytole tetraacrylate polymer. In the insert: enlarged chromatograms of extracted samples between 2.4 and 2.7 min.

Satisfactory sample clean-up was achieved through the application of the extraction protocol, as can be seen in Figure 3, where the chromatograms of apple juice spiked at 25, 50 and 100 μg L^−1^ of patulin are reported. By the comparison of the chromatograms it can be seen that 100 μg L^−1^ of patulin can be detected with difficulty when a sample of apple juice is separated directly by RP-HPLC without preliminary solid-phase extraction, while the same spiked sample analyzed after solid-phase extraction shows a clean chromatographic pattern, with the peak of patulin easily detected and, as a consequence, quantified.

Recoveries and repeatability were measured at three concentration levels of patulin appearing to be 64 ± 12% at 25 μg L^−1^; 83 ± 5.6% at 50 μg L^−1^; and 76 ± 4.5% at 100 μg L^−1^. Recoveries are fully in accordance with the performance criteria published by the European Union [21], except for the average value at 25 μg L^−1^ that exhibited results slightly under the recommended level of 70%. 

Thus, they were fairly reproducible and in good agreement with the recoveries performed directly on the citrate buffer. The within-day and between-day reproducibility was evaluated on extracted apple juice samples spiked with patulin at level of 25 μg L^−1^ in five replicates within a day and over the course of five consecutive days. The within-day and between-day reproducibility was 5.6% and 7.6%, respectively. 

## 3. Conclusions

This work showed that it is possible to identify a patulin-binding polymer by means of the screening of a 256-member combinatorial polymeric library, provided that the molecular diversity of such a library will be significantly high in terms of monomers, cross-linkers, and polymerization conditions considered. Finally, as a proof of concept, one of the best binding polymers identified by the screening protocols in acidic buffer and acetonitrile was successfully used as a solid-phase extraction material for clean-up and extraction of patulin from apple juice with fairly good results. To conclude, this approach demonstrated its usefulness in the selection of new kinds of polymeric materials for the development of solid-phase extraction protocol mainly in those cases where matrix complexity places severe limits on the performance of the analytical method of analysis.

## 4. Materials and Methods 

### 4.1. Materials 

2,2-Dimethoxy-2-phenylacetophenone (DMPA), all functional monomers (acrylamide, AM; acrylonitrile, AN; 4-acryloylmorpholine, AMO; allylamine, ALA; *N*,*N*-diethylaminoethylmethacrylate, DEAEM; *N*,*N*-dimethylacrylamide, DMAM; *N*,*N*-dimethylaminoethylmethacrylate, DMAEM; ethylenglycol methacrylate phosphate, EGMP; 2-hydroxyethylmethacrylate, HEMA; methacrylic acid, MAA; methylacrylate, MA; monomethoxypolyethylenglycol 400 methacrylate, PEGMA; styrene, STY; 1-vinylimidazole, VIM; 4-vinylpyridine, 4VP; *N*-vinylpyrrolidone, NVP), cross-linkers (ethylene dimethacrylate, EDMA; glycerol dimethacrylate, GDMA; pentaerithrytole tetraacrylate, PETRA; trimethylolpropane trimethacrylate, TRIM) were from Sigma (Milan, Italy). Polymerization inhibitors eventually present in monomer solutions were removed by clean-up on activated alumina columns. Acetic acid, all organic solvents and all other chemicals were from VWR International (Milano, Italy). All the solvents were of HPLC grade, whereas all chemicals were of analytical grade. Patulin was from Fermentek (Jerusalem, Israel).

Patulin stock solutions were prepared by dissolving 10.0 mg of solid mycotoxin in 10 mL of acetonitrile and stored in the dark at −20 °C until use. Apple juice certified as free from patulin was a gift from Generon s.r.l. (Modena, Italy).

### 4.2. Polymeric Combinatorial Library 

The polymeric combinatorial library was made up by 256 different polymer combinations reported in Table 1, Table 2, Table 3 and Table 4. In 3-mL thick wall borosilicate glass vials, the pre-polymerization solutions with a molar ratio of 1:9 between the functional monomer and the cross-linker were prepared by mixing 0.15 mmoles of functional monomer and 1.35 mmoles of cross-linker sampled by weight. Then, a volume of dry porogenic solvent corresponding to the total volume of the monomers and with a proper amount of DMPA (1% of the vinyl groups in the pre-polymerization mixture) was added. The vials were sonicated in an ultrasonic bath for 10 min and sealed. Then, the mixtures were photo-polymerized overnight at 4 °C by using a 200 W medium-pressure mercury lamp. The obtained bulk polymers were broken with a steel spatula, crushed in a mechanical mortar, suspended in ethanol, mechanically wet-sieved to 15–38 μm and finally dried under vacuum at 70 °C for 2 h. Finally, 50 mg of each polymer were packed in a 2-mL SPE empty polypropylene cartridge and subsequently mounted on the rack of a VersaPlateTM 96-well SPE system (Agilent, Milano, Italy). The cartridges were sequentially washed with 5 × 500 μL of water, 5 × 500 μL of methanol-acetic acid 1:9 (*v*/*v*) and 5 × 500 μL of acetonitrile, dried under a gentle stream of nitrogen for 2 h, sealed and stored at room temperature.

### 4.3. Library Screening 

Before each measurement, the polymeric combinatorial library was equilibrated with 5 × 500 μL of acetonitrile or citrate buffer 50 mM, pH 3.2. Subsequently, 200 μL of 2 mg L^−1^ solution of patulin in acetonitrile or citrate buffer were loaded into the cartridges and vacuum was applied to facilitate the passage of the solution through the polymer. The eluates were collected, transferred to 1.5-mL HPLC autosampler vials, and the unretained (free, F) patulin was measured by HPLC (vide infra). To evaluate the reproducibility of the screening assay, each elution was repeated three times and the amount of free patulin was estimated as the average of the measured values. The amount of patulin bound (bound, B) to the polymer was calculated by subtracting the amount of free patulin from the known initial amount (total, T). Statistical tests on the measured values were performed by using the statistical module of SigmaPlot 12.0 (Systat Software Inc., Richmond, CA, USA).

### 4.4. HPLC Analysis

Reverse phase HPLC analysis were performed on a LiChrosphere RP-18 125-4 (5 μm) from VWR (Milano, Italy). The HPLC apparatus was an Accela High Speed LC consisting of a quaternary solvent delivery pump, a thermostated autosampler provided with an injection system, a diode array UV detector and a data acquisition system Xcalibur 2.0, all from Thermo Fisher Scientific (Milano, Italy). The mobile phase was made of 30 + 70 (*v*/*v*) mixture of acetonitrile-ammonium acetate buffer 20 mM pH 5.0. The flow rate was set at 0.50 mL/min, the injection volume was 50 μL, and the detection wavelength was 276 nm. Patulin standard solutions at concentrations of 10, 25, 50, 100, 250, 500, and 1000 μg L^−1^ were prepared in acetonitrile-ammonium acetate buffer immediately before use. They were analyzed three times consecutively and peak areas were plotted against concentration. The calibration plot was drawn by using a weighted linear regression (weight = 1/conc, *r*^2^ = 0.9995). The limit of detection (LOD = 7.06 μg L^−1^) and the limit of quantification (LOQ = 21.4 μg L^−1^) were calculated as LOD = 3.3 Sy/b and LOQ = 10 Sy/b, where Sy is the standard error of the response and b is the slope of the calibration plot. 

### 4.5. Solid-Phase Extraction of Apple Juice

An adequate amount of polymer (100 mg) was suspended in acetonitrile, sonicated for 10 min in a water-bath and packed in 3-mL empty polypropylene SPE cartridges mounted on a SPE vacuum manifold (Macherey-Nagel, Duren, Germany). Immediately before use, the cartridge was activated with 3 × 1 mL of citrate buffer. When necessary, the cartridge was cleaned and regenerated by washing with 3 × 1 mL of acetonitrile—acetic acid (1:1 *v*/*v*).

Apple juice samples were centrifuged at 8000 rpm for 15 min and filtered through 0.22-μm polypropylene membranes. The filtered solution was spiked with patulin to a final concentration of 25, 50 or 100 μg L^−1^ and immediately extracted by loading 2 mL into the activated cartridge and applying vacuum to flow the sample easily through the stationary phase. After sample loading, air was passed through the column for 10 min. Then, the cartridge was sequentially washed with 0.5 mL of citrate buffer, with 0.5 mL of ultrapure water, and again dried by passing air for 5 min. Patulin was recovered by eluting the cartridge with 3 × 0.5 mL of acetonitrile; the organic solution was then evaporated under a gentle stream of nitrogen and re-dissolved under sonication in 0.2 mL of acetonitrile. To evaluate the reproducibility of the SPE protocol, each extraction was repeated five times and patulin recovery was evaluated as the average of the single values measured.

## Figures and Tables

**Table 1:** Patulin binding in buffer and acetonitrile for polymers prepared in toluene. Data in bold italics: B/T ≤ 0.05 or B/T ≥ 0.95. Underlined data pairs: B/T ≥ 0.95 in buffer and B/T ≤ 0.05 in acetonitrile.

Cross-Linker/Monomer	EDMA		GDMA		TRIM		PETRA	
	Buffer	MeCN	Buffer	MeCN	Buffer	MeCN	Buffer	MeCN
MAA	***1.00***	0.51	***1.00***	0.43	***1.00***	***0.02***	***1.00***	0.13
DMAEM	***1.00***	0.43	0.73	0.64	0.93	0.47	***0.99***	***0.02***
EGMP	***0.98***	0.45	0.74	0.50	***1.00***	0.31	***0.99***	0.14
ALA	***1.00***	0.51	0.84	0.50	***0.99***	0.40	***0.99***	0.50
DEAEM	***1.00***	0.39	0.69	0.43	***0.97***	0.37	***0.97***	0.22
4VP	***1.00***	0.51	0.87	0.26	***1.00***	0.14	***0.99***	0.23
VIM	***1.00***	0.37	0.77	0.31	***1.00***	***0.01***	0.93	0.27
AM	***1.00***	0.43	0.82	0.76	***1.00***	0.19	***0.98***	0.46
AN	***1.00***	***0.04***	0.71	0.09	***1.00***	0.30	***1.00***	0.39
DMA	***1.00***	0.51	0.76	0.38	***1.00***	0.56	***1.00***	0.25
HEMA	***1.00***	***0.00***	0.83	0.43	***1.00***	0.25	0.93	0.29
MMA	***1.00***	***0.00***	0.63	0.50	***1.00***	0.39	***1.00***	0.39
STY	***1.00***	***0.00***	0.79	0.21	***1.00***	0.19	***0.99***	0.28
ACM	***1.00***	***0.04***	0.76	***0.05***	***1.00***	0.20	***1.00***	0.43
VPO	***1.00***	0.58	0.75	0.27	***1.00***	0.62	***1.00***	0.43
PEGMA	***1.00***	0.66	0.71	0.16	***1.00***	***0.03***	***1.00***	0.41

**Table 2:** Patulin binding in buffer and acetonitrile for polymers prepared in acetonitrile. Data in bold italics: B/T ≤ 0.05 or B/T ≥ 0.95. Underlined data pairs: B/T ≥ 0.95 in buffer and B/T ≤ 0.05 in acetonitrile.

Cross-Linker/Monomer	EDMA		GDMA		TRIM		PETRA	
	Buffer	MeCN	Buffer	MeCN	Buffer	MeCN	Buffer	MeCN
MAA	***1.00***	0.11	0.88	0.09	***0.99***	0.38	***1.00***	0.47
DMAEM	0.91	0.41	0.79	***0.02***	***1.00***	***1.00***	***1.00***	0.56
EGMP	***1.00***	0.09	0.85	0.75	0.92	0.78	***1.00***	0.10
ALA	***1.00***	***0.01***	0.92	0.43	***1.00***	0.34	***1.00***	0.71
DEAEM	***1.00***	0.14	***0.96***	0.52	***1.00***	0.61	***1.00***	0.34
4VP	***1.00***	0.68	0.78	0.27	0.89	0.77	***1.00***	0.32
VIM	***0.97***	0.48	0.89	***0.01***	***1.00***	0.43	***1.00***	0.59
AM	***0.99***	0.14	0.88	0.70	***1.00***	0.49	***1.00***	0.50
AN	***1.00***	0.44	0.92	0.35	***0.99***	0.53	***1.00***	0.41
DMA	***1.00***	0.58	***0.96***	0.49	***1.00***	0.52	***1.00***	0.40
HEMA	***1.00***	0.41	0.84	0.42	***1.00***	0.54	***0.98***	0.46
MMA	***1.00***	0.60	***0.95***	0.48	***1.00***	0.39	***1.00***	0.42
STY	***1.00***	0.45	0.88	0.39	***1.00***	0.48	***1.00***	0.40
ACM	***1.00***	0.54	0.82	0.40	***1.00***	0.46	***1.00***	0.39
VPO	***1.00***	0.53	0.91	0.44	***1.00***	0.52	***1.00***	0.50
PEGMA	***1.00***	0.42	0.84	0.39	***1.00***	0.27	***1.00***	0.40

**Table 3:** Patulin binding (bound to total, B/T) in buffer and acetonitrile for the combinatorial library of polymers prepared in chloroform. Data in bold italics: polymers with B/T ≤ 0.05 or B/T ≥ 0.95. Underlined data pairs: polymers with B/T ≥ 0.95 in buffer and B/T ≤ 0.05 in acetonitrile.

Cross-Linker/Monomer	EDMA		GDMA		TRIM		PETRA	
	Buffer	MeCN	Buffer	MeCN	Buffer	MeCN	Buffer	MeCN
MAA	***1.00***	0.34	0.91	0.17	***1.00***	0.29	***1.00***	***0.00***
DMAEM	***1.00***	0.71	0.94	0.35	***0.99***	0.26	***1.00***	0.46
EGMP	***0.99***	***0.00***	0.83	0.22	0.91	***0.00***	***1.00***	0.27
ALA	***0.96***	***0.00***	0.82	0.17	***1.00***	0.42	***1.00***	***0.01***
DEAEM	***1.00***	0.64	***0.95***	0.68	***1.00***	0.39	***0.99***	0.14
4VP	0.90	0.58	0.93	0.38	***0.98***	0.60	***1.00***	0.30
VIM	***0.98***	***0.00***	***0.95***	0.53	***1.00***	0.82	***1.00***	***0.00***
AM	***1.00***	0.48	***0.97***	0.36	0.93	***0.03***	***1.00***	0.10
AN	***1.00***	***0.00***	0.90	0.50	***1.00***	***1.00***	***1.00***	***0.05***
DMA	***1.00***	***0.01***	***1.00***	***1.00***	***1.00***	0.71	***1.00***	0.15
HEMA	***1.00***	0.49	0.82	0.67	0.87	0.67	***1.00***	***1.00***
MMA	***1.00***	0.43	***0.98***	***0.00***	***1.00***	***1.00***	***1.00***	***1.00***
STY	***1.00***	***0.00***	***0.95***	***1.00***	0.93	0.86	***1.00***	0.39
ACM	***1.00***	0.47	***0.95***	***1.00***	***0.95***	***0.00***	***1.00***	***1.00***
VPO	***1.00***	0.60	***1.00***	0.33	***1.00***	***1.00***	***1.00***	0.13
PEGMA	0.94	0.35	0.92	0.70	0.85	0.85	***1.00***	0.47

**Table 4:** Patulin binding (bound to total, B/T) in buffer and acetonitrile for the combinatorial library of polymers prepared in tetrahydrofurane. Data in bold italics: polymers with B/T ≤ 0.05 or B/T ≥ 0.95. Underlined data pairs: polymers with B/T ≥ 0.95 in buffer and B/T ≤ 0.05 in acetonitrile.

Cross-Linker/Monomer	EDMA		GDMA		TRIM		PETRA	
	Buffer	MeCN	Buffer	MeCN	Buffer	MeCN	Buffer	MeCN
MAA	***1.00***	0.33	0.92	0.14	***1.00***	0.51	0.93	***0.00***
DMAEM	***1.00***	0.62	***0.97***	0.38	***1.00***	0.88	***1.00***	0.41
EGMP	***1.00***	0.52	0.91	0.15	***1.00***	0.39	***1.00***	***0.00***
ALA	***1.00***	0.24	0.88	0.19	***1.00***	0.45	***0.99***	***0.00***
DEAEM	***1.00***	0.72	0.85	0.25	***1.00***	0.69	***1.00***	***0.00***
4VP	***1.00***	0.58	***0.98***	0.25	***1.00***	0.46	0.92	***0.00***
VIM	***1.00***	0.84	***0.98***	0.40	***1.00***	0.73	***0.99***	0.46
AM	***1.00***	0.28	***0.96***	0.18	***1.00***	0.32	***0.99***	***0.00***
AN	***1.00***	0.22	***0.97***	0.15	***1.00***	0.63	***1.00***	0.40
DMA	***1.00***	0.49	***0.99***	0.34	***0.97***	0.51	***1.00***	0.50
HEMA	***0.98***	0.43	***1.00***	0.35	***1.00***	0.37	***0.99***	0.61
MMA	***0.99***	***0.00***	***0.99***	0.70	***1.00***	0.51	***0.99***	0.17
STY	***1.00***	0.28	***1.00***	0.69	***1.00***	0.70	***1.00***	***1.00***
ACM	***1.00***	0.52	***1.00***	0.91	***1.00***	0.52	***1.00***	0.55
VPO	***0.99***	0.43	***0.95***	0.60	***1.00***	0.09	***1.00***	0.58
PEGMA	***1.00***	***1.00***	0.86	0.25	***1.00***	0.18	***1.00***	0.12
